# A Bezoar Composed of Bilirubin Calcium, Calcium Carbonate, and Fatty Acid Calcium

**DOI:** 10.1155/2019/5742672

**Published:** 2019-05-23

**Authors:** Masaya Iwamuro, Haruo Urata, Shoichiro Hirata, Toru Ueki, Tetsuro Hanabata, Sho Takeda, Akira Teraoka, Hiroyuki Okada

**Affiliations:** ^1^Department of Gastroenterology and Hepatology, Okayama University Graduate School of Medicine, Dentistry, and Pharmaceutical Sciences, Okayama 700-8558, Japan; ^2^Department of Internal Medicine, Teraoka Memorial Hospital, Fukuyama 729-3103, Japan; ^3^Central Research Laboratory, Okayama University Medical School, Okayama 700-8558, Japan; ^4^Department of Internal Medicine, Fukuyama City Hospital, Fukuyama 721-8511, Japan; ^5^Department of Surgery, Teraoka Memorial Hospital, Fukuyama 729-3103, Japan; ^6^Department of Neurosurgery, Teraoka Memorial Hospital, Fukuyama 729-3103, Japan

## Abstract

A 68-year-old Japanese man was diagnosed with bezoar in the stomach, which was endoscopically retrieved. The bezoar was composed of bilirubin calcium, calcium carbonate, and fatty acid calcium. Due to the presence of bilirubin calcium in the bezoar, we performed imaging studies of the bile duct; gallstones and common bile duct stones were identified. Although bezoar with components similar to bile is infrequently encountered, our findings suggest that a bezoar originating from bile should be considered among the differential diagnoses in patients without a recent consumption history of persimmons who demonstrate a mass in the digestive tract. This case highlights the importance of component analysis of gastric bezoars because its findings may alter the treatment plan.

## 1. Introduction

Bezoars are solid masses of inedible or undigested material accumulated in the gastrointestinal tract. Although bezoars can exist in any part of the digestive tract, they most commonly occur in the stomach [1, 2]. Bezoars are classified according to their composition; among them, phytobezoars comprising plant materials, such as fibers, skin, and seeds of vegetables and fruits, are the most common. In particular, bezoars formed after consumption of persimmon, i.e., diospyrobezoar, account for a large percentage of bezoars found in Japan [2–4]. Other types of bezoars include trichobezoars (ingested hair), pharmacobezoars (medications such as sucralfate and aluminum hydroxide gel), and lactobezoars (milk protein). Although various kinds of materials have been reported as a component of bezoars, bezoars having a composition similar to that of gallstones and bile duct stones are quite infrequently encountered.

We recently encountered a patient with a bezoar in the stomach. Based on the results of infrared spectroscopy, we determined that the bezoar consisted of fatty acid calcium, calcium carbonate, and bilirubin calcium. The detection of the bilirubin calcium component led us to identify cholecystolithiasis and common bile duct stones. This case highlights the importance of component analysis of gastric bezoars because its findings may alter the treatment plan.

## 2. Materials and Methods

A 68-year-old Japanese man presented with intermittent abdominal pain and appetite loss. The patient had noticed epigastric pain five years earlier, and the abdominal pain had started when he was working as a woodworker. He also mentioned that his body weight had decreased by 5 kg during the last three years. The patient had not been taking any medication and denied consuming nutritional supplements or herbal medicines. Interviews with the patient revealed no consumption of persimmon for at least 5 years. He had received treatment for sinusitis at 16 years of age and for tooth decay at 66 years of age; however, he had no history of gastrointestinal disease. His body temperature was 37.7°C and blood pressure was 142/88 mmHg. Physical examination revealed no abnormalities, including within the abdomen. Laboratory tests revealed increased levels of white blood cells (14,900/*μ*L), C-reactive protein (22.94 mg/dL), alkaline phosphatase (499 IU/L), gamma glutamyl transpeptidase (194 IU/L), total bilirubin (1.5 mg/dL), and direct bilirubin (0.8 mg/dL). Aspartate aminotransferase and alanine aminotransferase levels were not elevated. Hemoglobin (12.9 g/dL) and hematocrit (39.3%) levels were decreased, whereas red blood cells were within the normal ranges.

**Figure 1:**
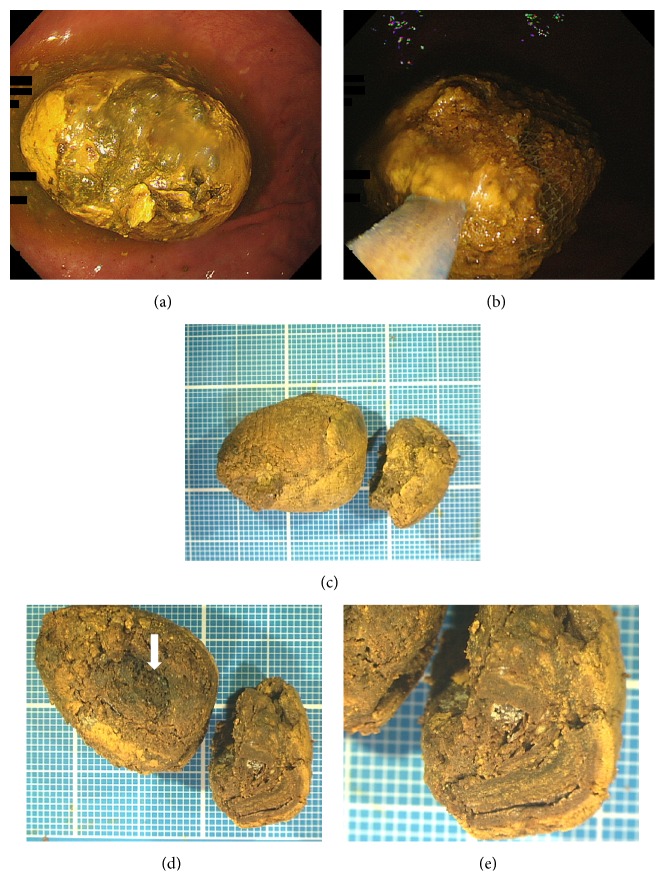
Images of a gastric bezoar. Esophagogastroduodenoscopy revealed a bezoar in the stomach (a), which was endoscopically retrieved (b). The surface of the bezoar was yellowish (c), and the cut surface showed laminar structure (d, e). Each grid section is a 1 mm square (c-e).

**Figure 2:**
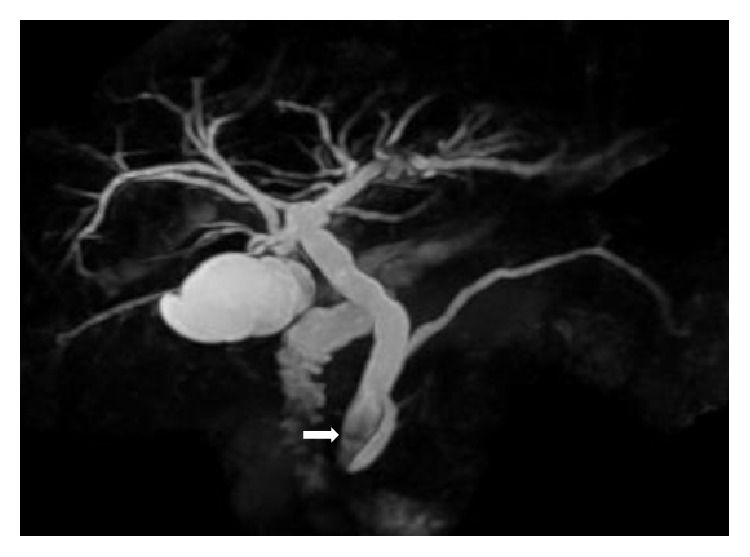
Magnetic resonance cholangiopancreatography image. Radiology examinations revealed gall stones and common bile duct stones (arrow), but no fistulas.

**Figure 3:**
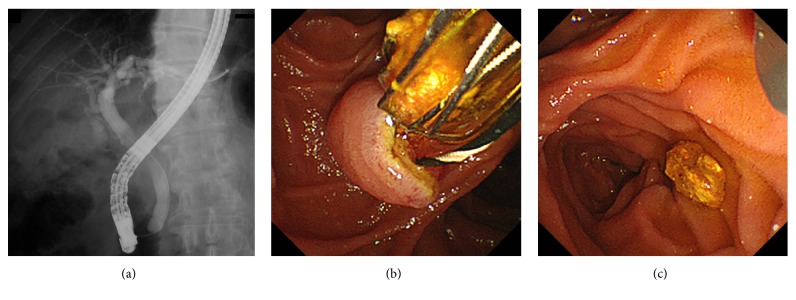
Endoscopic retrograde cholangiography images. Cholangiogram revealed no fistulas or anomalies (a). Small stones were endoscopically retrieved from the common bile duct (b, c).

Esophagogastroduodenoscopy revealed a yellowish bezoar, measuring approximately 4 cm in diameter, in the stomach (Figure 1(a)). The bezoar was endoscopically retrieved using a net device (Figures 1(b) and 1(c)). The cut surface of the bezoar showed laminar structure with a concentric ring pattern resembling a tree trunk (Figures 1(d) and 1(e)). As described below, the component analysis revealed the presence of bilirubin calcium in the retrieved bezoar. Based on the bilirubin calcium component and the increased levels of white blood cells, C-reactive protein, alkaline phosphatase, gamma-glutamyl transpeptidase, total bilirubin, and direct bilirubin, we suspected bile duct stones. Therefore, we performed computed tomography (CT) and magnetic resonance cholangiopancreatography. Radiology examinations revealed gall stones and common bile duct stones, but no fistulas (Figure 2). Subsequently, endoscopic retrograde cholangiography and endoscopic sphincterotomy were performed. Cholangiography revealed a small defect in the common bile duct, suggesting bile stones. No fistulas or anomalies were noted on a cholangiogram (Figure 3(a)). Small, yellow fragments of bile duct stones were endoscopically retrieved by a snare device (Figures 3(b) and 3(c)). The patient underwent cholecystectomy six weeks after the lithotomy. However, no stones were detected in the surgically removed gallbladder.

The bezoars and common bile duct stones were used in the subsequent analysis. Infrared spectroscopy was performed for the bezoar by SRL Inc. (Tokyo, Japan [5]); however, an analysis could not be performed of the common bile duct stones because the two small stone pieces that were retrieved endoscopically were subjected to scanning electron microscopy analysis. We performed an energy-dispersive X-ray spectrometry (EDX) analysis, as previously described [6]. The sample was air-dried and the surface was coated with osmium for 10 seconds (HPC-1S-type osmium coater; Shinku Device Co., Ibaraki, Japan). To investigate the elements contained within the bezoar, an EDX analysis was performed using an S4800 scanning electron microscope (Hitachi, Tokyo, Japan) with an accelerating voltage of 25 kV and an EDAX Genesis APEX2 EDX system (AMETEK Inc., Paoli, PA, USA). Scanning electron microscopy images were captured using an S4800 electron microscope. Commercially available bilirubin powder (Sigma-Aldrich, St. Louis, MO, USA) was also analyzed using EDX and an electron microscope.

## 3. Results

**Figure 4:**
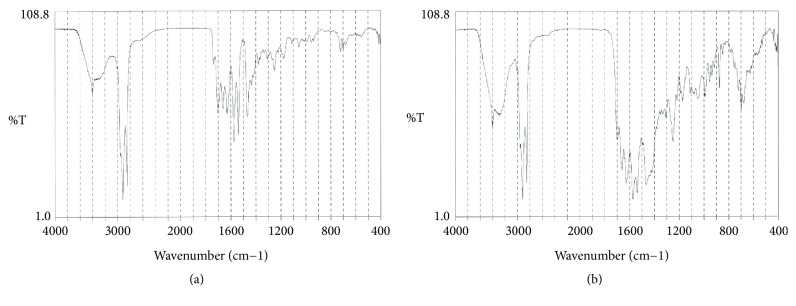
Infrared spectroscopy images. Infrared spectroscopy revealed that the external layer of the bezoar was composed of fatty acid calcium (61%) and bilirubin calcium (39%) (a). The core of the bezoar was composed of bilirubin calcium (48%), calcium carbonate (36%), and fatty acid calcium (16%) (b).

**Figure 5:**
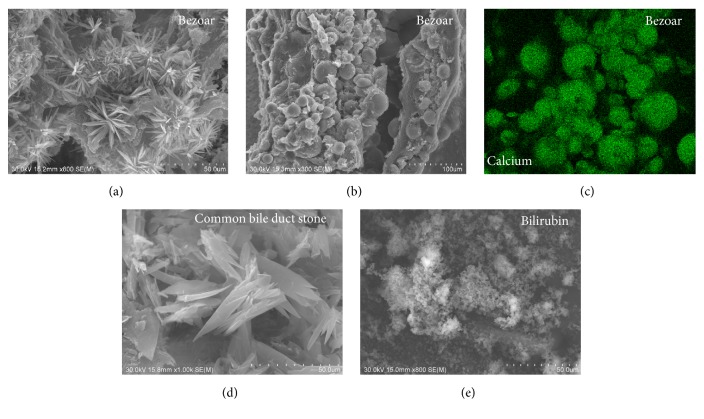
Electron microscopy images. The cut surface of the bezoar showed acicular crystals (a). Multiple globular substances were observed only in the bezoar core (b). Elemental mapping showed a large amount of calcium deposition in the globular substances (c). The common bile duct stones also showed acicular crystals (d). However, the width and length of these stones were greater than those of the bezoar. Crystals were not observed in the commercially available bilirubin powder (e).

Infrared spectroscopy revealed that the external layer of the bezoar was composed of fatty acid calcium (61%) and bilirubin calcium (39%) (Figure 4(a)). In contrast, the core of the bezoar, which appeared brownish (Figure 1(d), arrow), was composed of bilirubin calcium (48%), calcium carbonate (36%), and fatty acid calcium (16%) (Figure 4(b)). Electron microscopic observation of the cut surface of the bezoar showed acicular crystals (Figure 5(a)). Multiple globular substances were observed only in the bezoar core (Figure 5(b)). Elemental mapping showed a large amount of calcium deposition in the globular substances (Figure 5(c)). Although the common bile duct stones also showed acicular crystals, their width and length were greater than those of the bezoar, resembling carving knives (Figure 5(b)). Crystals were not observed in bilirubin powder (Figure 5(c)).

**Figure 6:**
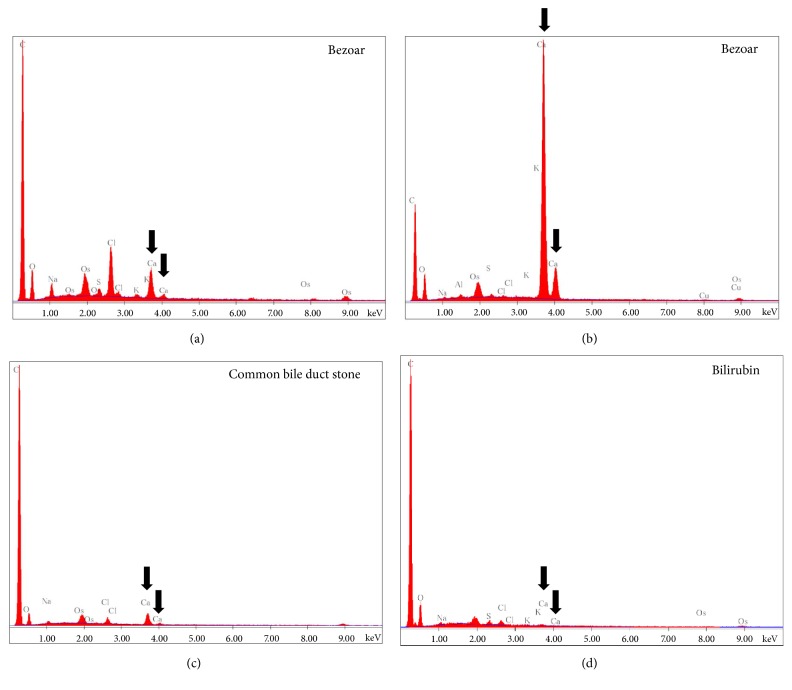
Results of EDX analysis of the bezoar's external layer (a), the bezoar's core (b), common bile duct stones (c), and commercially available bilirubin powder (d). Arrows indicate calcium.

EDX analysis of the bezoar's external layer showed high concentrations of carbon, followed by chlorine, calcium, oxygen, sodium, along with a small amount of sulfur and potassium (Figure 6(a)). The bezoar's core had a significantly higher concentration of calcium and lower concentrations of carbon, sodium, and chlorine than the external layer (Figure 6(b)). The common bile duct stones were composed of carbon, calcium, oxygen, chlorine, and sodium (Figure 6(c)). The relative concentrations of calcium, oxygen, chlorine, and sodium were smaller than those of the bezoar. Sulfur and potassium were not detected in the common bile duct stones. In contrast, bilirubin powder contained no or small amount of calcium (Figure 6(d)). The main elemental composition of bilirubin powder was carbon, along with a small amount of oxygen.

## 4. Discussion

Gallstones are classified into cholesterol stones, brown stones, black stones, and mixed stones [7–9]. The white stones are composed of ≥50% of cholesterol, whereas black and brown stones, which contain different proportions of cholesterol and bilirubin, are composed of ≤30% of cholesterol by weight [10]. Gallstone components other than cholesterol and bilirubin include calcium carbonate, phosphate salts, phospholipids, fatty salts, polysaccharide, and proteins, along with minor elements such as potassium, calcium, manganese, iron, copper, zinc, chlorine, and sulfur [7, 11–14]. In the present patient, infrared spectroscopy revealed that the bezoar was composed of fatty acid calcium, calcium carbonate, and bilirubin calcium.

As described previously, persimmon phytobezoars, i.e., diospyrobezoars, are the most common type of bezoars in Japan [3]. Meanwhile, gastric bezoars with components similar to gallstones are quite rare. To the best of our knowledge, only one patient with such a bezoar has been reported [15]. Nawata et al. reported a 70-year-old Japanese woman who presented with epigastric pain. Fluoroscopy examination revealed a round, mobile mass in the stomach. However, esophagogastroduodenoscopy performed one day after fluoroscopy revealed no mass in the esophagus, stomach, and duodenum, and the patient developed ileus. Laparotomy was performed, and a dark green bezoar was removed from the ileum. Infrared spectroscopy revealed that the bezoar was composed of bilirubin calcium and cholesterol.

In the present case, infrared spectroscopy revealed that fatty acid calcium and bilirubin calcium existed in the external layer and bilirubin calcium, calcium carbonate, and fatty acid calcium were contained in the core. Electron microscopy showed multiple globular substances in the bezoar's core, in which a large amount of calcium was deposited. Although the exact material forming the globular substances could not be identified, we believe that it could be carbonate bioliths formed by a bacterial strain [16, 17]. Calcium carbonate is a food additive, and its mineral components can be produced by some bacterial strains through mineral bioprecipitation. Fatty acids are produced by human pancreatic lipase, which hydrolyzes dietary fat molecules in the digestive tract, converting ingested oils to monoglycerides and fatty acids. Therefore, we speculate that calcium carbonate and fatty acids that were supplied from the patient's diet and possibly bacteria were involved in the bezoar's formation.

Several mechanisms underlying the pathogenesis of gastric bezoar formation that has components similar to bile or gallstones can be hypothesized. First, bile excreted from the bile duct to the duodenum might flow into the stomach. A chemical substance that constitutes bile, such as bilirubin calcium, cholesterol, and fatty acid calcium, might precipitate, causing bezoar formation in the stomach. Second, small bile stones might be spontaneously discharged from the bile duct via the major duodenal papilla. These stones might flow into the stomach, accumulate, and form a bezoar. Third, epitaxy, the process of growing a crystal of a particular orientation atop another crystal in which the orientation is determined by the underlying crystal, might occur. Bile duct stones might flow into the stomach and continue to grow with the precipitation of bile. Bilirubin calcium and fatty acid calcium could precipitate and result in epitaxial growth on the bile duct stones and larger crystal formation. Fourth, bile or gallstones might be directly excreted to the stomach through a fistula between the stomach and the gall bladder or the bile duct, leading to bezoar formation. However, no fistula was detected in the present patient and the previously reported patient [15]. In the present patient, scanning electron microscopy analysis revealed that the crystals of the gastric bezoar were different in size from those of the bile duct stones. Therefore, we speculate that epitaxial growth occurred with the precipitation of bilirubin calcium and fatty acid calcium. We also speculate that bile flowed into the stomach from the duodenum, and precipitation of bilirubin calcium and fatty acid calcium resulted in bezoar formation. Because microstructural observation was performed in only a single patient in the present study, further analyses of other cases would reveal the mechanisms of development of bezoars that originate from bile.

Component analysis of bezoars is essential to preventing their recurrence. For example, in patients with diospyrobezoars, the avoidance of persimmon consumption is proactively recommended. The administration of prokinetic agents could effectively prevent phytobezoars since the reduced evacuation of indigestible foods due to insufficient gastric motor activity can lead to bezoar formation [2]. In the present case, the presence of bilirubin calcium in the retrieved bezoar prompted us to perform CT and magnetic resonance cholangiopancreatography, which led to the identification of bile stones.

In conclusion, we report a case of a gastric bezoar containing bilirubin calcium, calcium carbonate, and fatty acid calcium. Although this was a rare presentation, our findings suggest that a bezoar originating from bile should be considered among the differential diagnoses in patients without a recent consumption history of persimmons who demonstrate a mass in the digestive tract. In addition, this case underscores the importance of component analysis of gastric bezoars because it can reveal underlying diseases such as bile stones as described here.

## References

[1] Sanders M. K. (2004). Bezoars: from mystical charms to medical and nutritional management. *Practical Gastroenterology*.

[2] Iwamuro M., Okada H., Matsueda K. (2015). Review of the diagnosis and management of gastrointestinal bezoars. *World Journal of Gastrointestinal Endoscopy*.

[3] Iwamuro M., Tanaka S., Shiode J. (2014). Clinical characteristics and treatment outcomes of nineteen Japanese patients with gastrointestinal bezoars. *Internal Medicine*.

[4] Iwamuro M., Tanaka S., Moritou Y. (2017). Importance of second-look endoscopy on an empty stomach for finding gastric bezoars in patients with gastric ulcers. *Acta Medica Okayama*.

[5] Shareef K. M., Omer L. A., Garota S. A. (2008). Predicting the chemical composition of gallstones by FTIR spectroscopy. *Biomedical & Pharmacology Journal*.

[6] Iwamuro M., Miyashima Y., Yoshioka T. (2014). Ultrastructural analysis of an enterolith composed of Deoxycholic acid. *Acta Medica Okayama*.

[7] Cavalu S., Popa A., Bratu I., Borodi G., Maghiar A. (2015). New evidences of key factors involved in “silent stones” etiopathogenesis and trace elements: microscopic, spectroscopic, and biochemical approach. *Biological Trace Element Research*.

[8] Chowdhury A. H., Lobo D. N. (2011). Gallstones. *Surgery*.

[9] Trotman B. W. (1991). Pigment gallstone disease. *Gastroenterology Clinics of North America*.

[10] Cariati A. (2015). Gallstone classification in western countries. *Indian Journal of Surgery*.

[11] Alatise O. I., Obiajunwa E. I., Lawal O. O., Adesunkanmi A. R. K. (2010). Particle-induced x-ray emission (PIXE) analysis of minor and trace elements in gallstones of Nigerian patients. *Biological Trace Element Research*.

[12] Iordanidis A., Garcia-Guinea J., Giousef C., Angelopoulos A., Doulgerakis M., Papadopoulou L. (2013). Characterization of Gallbladder stones from cholelithiasis patients of northern greece, using complementary techniques. *Spectroscopy Letters*.

[13] Kaloustian J., De La Porte P. L., El-Moselhy T., Lafont H., Portugal H. (2005). Thermal analysis and microscopical characterization of cholesterol in gallstones. *Journal of Thermal Analysis and Calorimetry*.

[14] Gümüş M., Yüksel H., Evliyaoğlu O. (2011). Effects of ellagic acid on copper, zinc, and biochemical values in serum and liver of experimental cholestatic rats. *Biological Trace Element Research*.

[15] Nawata Y., Mori F., Kurata S., Morita T., Kaneyuki T. (1979). A case of intestinal obstruction with bezoar. *The Japanese Journal of Gastroenterological Surgery*.

[16] Gonzalez-Martinez A., Leyva-Díaz J. C., Rodriguez-Sanchez A. (2015). Isolation and metagenomic characterization of bacteria associated with calcium carbonate and struvite precipitation in a pure moving bed biofilm reactor-membrane bioreactor. *Biofouling*.

[17] Rivadeneyra M. A., Ramos-Cormenzana A., Delgado G., Delgado R. (1996). Process of carbonate precipitation by Deleya halophila. *Current Microbiology*.

